# Elevated HbA_1c_ levels and the accumulation of differentiated T cells in CMV^+^ individuals

**DOI:** 10.1007/s00125-015-3731-4

**Published:** 2015-08-20

**Authors:** Jerrald L. Rector, G. Neil Thomas, Victoria E. Burns, Jennifer B. Dowd, Raphael M. Herr, Paul A. Moss, Marc N. Jarczok, Kristina Hoffman, Joachim E. Fischer, Jos A. Bosch

**Affiliations:** School of Sport, Exercise, and Rehabilitation Sciences, University of Birmingham, Birmingham, UK; Mannheim Institute of Public Health, Social and Preventive Medicine, Mannheim Medical Faculty, University of Heidelberg, Mannheim, Germany; School of Health and Population Sciences, College of Medical and Dental Sciences, University of Birmingham, Birmingham, UK; CUNY School of Public Health, New York, NY USA; CUNY Institute for Demographic Research, New York, NY USA; Cancer Research UK Centre, University of Birmingham, Birmingham, UK; Department of Psychology, University of Amsterdam, Weesperplein 4, 1018 XA Amsterdam, The Netherlands

**Keywords:** Cholesterol, CMV, Cytomegalovirus, Diabetes, Glucose, Haemoglobin A_1c_, HbA_1c_, Immune ageing, Metabolic syndrome, T cell

## Abstract

**Aims/hypothesis:**

Biological ageing of the immune system, or immunosenescence, predicts poor health and increased mortality. A hallmark of immunosenescence is the accumulation of differentiated cytotoxic T cells (CD27^−^CD45RA^+/−^; or dCTLs), partially driven by infection with the cytomegalovirus (CMV). Immune impairments reminiscent of immunosenescence are also observed in hyperglycaemia, and in vitro studies have illustrated mechanisms by which elevated glucose can lead to increased dCTLs. This study explored associations between glucose dysregulation and markers of immunosenescence in CMV^+^ and CMV^−^ individuals.

**Methods:**

A cross-sectional sample of participants from an occupational cohort study (*n* = 1,103, mean age 40 years, 88% male) were assessed for HbA_1c_ and fasting glucose levels, diabetes, cardiovascular risk factors (e.g. lipids), numbers of circulating effector memory (EM; CD27^−^CD45RA^−^) and CD45RA re-expressing effector memory (EMRA; CD27^−^CD45RA^+^) T cells, and CMV infection status. Self-report and physical examination assessed anthropometric, sociodemographic and lifestyle factors.

**Results:**

Among CMV^+^ individuals (*n* = 400), elevated HbA_1c_ was associated with increased numbers of EM (*B* = 2.75, *p* < 0.01) and EMRA (*B* = 2.90, *p* < 0.01) T cells, which was robust to adjustment for age, sex, sociodemographic variables and lifestyle factors. Elevated EM T cells were also positively associated with total cholesterol (*B* = 0.04, *p* < 0.05) after applying similar adjustments. No associations were observed in CMV^−^ individuals.

**Conclusions/interpretation:**

The present study identified consistent associations of unfavourable glucose and lipid profiles with accumulation of dCTLs in CMV^+^ individuals. These results provide evidence that the impact of metabolic risk factors on immunity and health can be co-determined by infectious factors, and provide a novel pathway linking metabolic risk factors with accelerated immunosenescence.

**Electronic supplementary material:**

The online version of this article (doi:10.1007/s00125-015-3731-4) contains peer-reviewed but unedited supplementary material, which is available to authorised users.

## Introduction

The progressive impairment of immunity with age, known as immunosenescence, is thought to underlie increased infection risk and mortality [1, 2] and may also contribute to several other age-associated complications, including low-grade inflammation and increased cardiovascular disease (CVD) risk [3–5]. Infection with cytomegalovirus (CMV) has been shown to accelerate features of immunosenescence [2, 6–8]. This herpes virus establishes a lifelong latent infection, interrupted by periods of non-clinical reactivation. The resultant activation of CMV-specific T cells leads to a marked accumulation of differentiated cytotoxic T cells (dCTLs; CD27^−^CD45RA^+/−^), which can be subdivided into effector memory (EM; CD27^−^CD45RA^−^) T cells and CD45RA re-expressing EM T cells (EMRA; CD27^−^CD45RA^+^) [6, 9]. Indeed, infected (i.e. CMV^+^) individuals have on average three- to fourfold higher dCTL numbers compared with uninfected (i.e. CMV^−^) individuals, although large inter-individual differences exist [10].

The accumulation of dCTLs may make a material contribution to the acceleration of immunosenescence [11] and is thought to provide a mechanism through which immunosenescence may be associated with health outcomes, such as CVD [12]. For example, these T cells show a high production of proinflammatory cytokines, have short telomeres and have an aberrant proliferative capacity [13, 14]. Thus, the accumulation of dCTLs may be the mechanism linking CMV with the hallmarks of immune ageing.

Significantly, many of the immune system impairments that have been associated with ageing resemble those of chronic hyperglycaemia. For example, impaired glucose tolerance and diabetes are associated with poor control of infection [15–17], impaired vaccination responses [18], elevated inflammatory activity [19] and shorter leucocyte telomere length [4, 20]. These observations raise the question of whether the immune effects of hyperglycaemia may, at least in part, involve the accumulation of dCTLs [21]. For example, in vitro studies show that strong T cell stimulation – similar to that which might be elicited by CMV reactivation – enhances cellular glucose uptake, which can lead to the accumulation of readily activated memory T cells that acquire resistance to cell death [22]. This presents a potential mechanism whereby hyperglycaemia may amplify the CMV-induced accumulation of dCTLs.

Therefore, the aim of the current study was to examine the relationship between glucose metabolism (i.e. HbA_1c_ and fasting glucose levels and diabetic status) and EM and EMRA T cell numbers in a large sample of CMV^+^ and CMV^−^ individuals. It was hypothesised that the effects of CMV infection on dCTL numbers would be enhanced in CMV^+^ individuals that show evidence of elevated glucose. Additionally, other factors associated with hyperglycaemia that may contribute to increased dCTL accumulation were also examined, including markers of dyslipidaemia (i.e. elevated circulating triacylglycerol and LDL-cholesterol [LDL-C], and lower HDL-cholesterol [HDL-C]) and elements of the metabolic syndrome [23].

## Methods

### Participants

The present study was conducted among employees (*n* = 1,103; 88% male; mean age 40 years [range 18–64 years]) of a large European airplane manufacturer in the south of Germany who took part in a voluntary company health check in 2011. Participant characteristics are presented in Table 1. Participants received a personalised comprehensive health report. All data were anonymised before analysis. This study was approved by the ethics committee of the Medical Faculty Mannheim, Heidelberg University. All participants gave written informed consent.

**Table 1:** Participant sociodemographic and lifestyle characteristics

Characteristic	Total	CMV status		*p* value
		Positive	Negative	
*n* (%)	1,103	400 (36)	703 (64)	
Age (years)	40.1 ± 11.0	41.5 ± 11.1	39.3 ± 10.8	<0.001
Sex (% male)	87.7	84.5	89.5	0.020
Married/co-habiting (%)	77.0	80.4	75.0	0.065
Job status (%)	–	–	–	0.007
Division/dept mgr	4.8	3.5	5.6	–
Project leader/process mgr	15.3	13.9	16.2	–
Worker (mgrl)	6.7	6.0	7.1	–
Skilled worker (non-mgrl)	63.8	62.6	64.4	–
Semi-skilled worker	9.4	14.1	6.7	–
Shift worker (% yes)	28.1	33.8	24.9	0.003
Manual occupation (% yes)	49.6	55.0	46.5	0.008
Smoking (%)	–	–	–	0.050
Never smoker	46.1	41.1	48.9	–
Former smoker	24.9	27.0	23.8	–
Smoker	29.0	31.9	27.3	–
Cigarettes per day (in smokers)	14 ± 8	15 ± 7	13 ± 8	0.090
Alcohol (%)	–	–	–	<0.001
0–2 times/month	24.9	30.4	21.7	–
1–2 times/week	29.9	32.5	28.5	–
3–7 times/week	45.2	37.2	49.8	–
Leisure physical activity (h/week)	7.0 ± 7.5	7.1 ± 9.4	7.0 ± 6.2	0.307

Data are unadjusted comparisons of participant characteristics: a Student’s *t* test was performed for continuous variables and a *χ* ^2^ test for categorical variables

Data are means ± SD unless otherwise stated

Dept, department; mgr, manager; mgrl, managerial

### Procedures

Participants arrived at a location away from their usual workplace between 06:45 and 08:45 hours in the morning for their health check. After drawing fasting venous blood and a medical examination, participants were seated in a quiet room to fill out questionnaires on demographic, medical and health behaviour data. Anthropometric (e.g. height, weight, waist and hip circumference) and BP measurements were carried out by trained study personnel. Demographic data, including age, sex and marital status, along with socioeconomic status (SES) indicators (measured as hierarchical job position, manual occupation and shift work), self- and doctor-diagnosed medical conditions, and lifestyle factors (e.g. smoking, alcohol intake, exercise) were obtained by questionnaires used and validated in the MONICA (‘Monitoring trends and determinants in cardiovascular disease’) study [24].

### Flow cytometry

T cell phenotypes were assessed by flow cytometry. Whole blood samples were collected in EDTA-coated tubes (Sarstedt, Nümbrecht, Germany), stored at room temperature and prepared within 1 h of collection. Briefly, 30 μl whole blood was stained with a combination of the following conjugated monoclonal antibodies: anti-CD3 allophycocyanin (APC)–cyanine dye 7 (Cy7; clone SK7), anti-CD4–peridinin chlorophyll protein (clone SK3), anti-γδ T cell receptor (γδTCR)–phycoerythrin (PE; clone B1), anti-CD8–APC (clone SK1; BD Biosciences, San José, CA, USA); and anti-CD45RA–FITC (clone HI100) and anti-CD27–PE-Cy7 (clone M-T271; BD Pharmingen, San Diego, CA). All antibodies were purchased from and validated by BD Biosciences and BD Pharmingen at pre-diluted concentrations for use at the recommended volume per test. Following a 20 min incubation at room temperature in the dark, 1.5 ml BD FACS lysing solution (BD Biosciences) was added to the mixture and incubated for another 15 min. After centrifugation for 7 min at 700 *g*, the supernatant was removed and both lysed erythrocytes and unbound antibody were washed away. The pellet was subsequently re-suspended in 250 μl 2% paraformaldehyde solution until analysis. Data were collected using a FACSCanto II flow cytometer and dedicated FACSDiva software (BD Biosciences). Spectral overlap was electronically compensated for using single labelled antibody tubes. Following data acquisition, files were transferred to a third party software program (FlowJo v7.6.5, Tree Star, Ashland, OR, USA) for analysis. Representative plots of the gating strategy are shown in electronic supplementary material Fig. 1. Lymphocyte numbers were obtained by multiplying the total leucocyte count by the percentage of gated lymphocytes. The lymphocyte number was further multiplied by the percentages of gated CD3^+^ cells and their subsequent subsets to calculate the numbers of cells per microlitre used in the analyses.

### CMV status determination

Fasting plasma samples were stored in small aliquots at −80°C until analysis. Evidence of a previous CMV infection (serostatus) was determined using a commercially available ELISA (BioCheck, Foster City, CA, USA) according to the manufacturer’s instructions. Optical density values obtained from participants’ samples were fitted to a standard curve. These concentrations were then compared with a cut-off value to compute CMV index scores. Participants with a borderline seropositive result, i.e. a calculated index score of >0.85 and <1.15, were re-tested (*n* = 9). If they remained borderline, participants with index scores above and below 1.00 were considered CMV^+^ and CMV^−^, respectively, as per the manufacturer’s instructions. The sensitivity, specificity and accuracy of the test are reported as 95.0%, 96.7% and 96.0%, respectively.

### Biochemical analysis

HbA_1c_, fasting glucose, triacylglycerol, LDL-C, HDL-C and high-sensitivity C-reactive protein levels were measured by an accredited clinical laboratory (Synlab Laboratories, Augsburg, Germany) according to standard laboratory procedures that comply with International Organization for Standardization norms (DIN EN ISO 15189). HbA_1c_ levels were measured using a second-generation HbA_1c_ immunoassay (Roche Diagnostics, Mannheim, Germany), and fasting glucose levels were measured using the glucose hexokinase enzymatic assay (Glucose OSR6121, Beckman Coulter, Brea, CA, USA), in accordance with the latest standardised guidelines and recommendations for laboratory analysis in the diagnosis of diabetes [25]. Cholesterol and triacylglycerol were automatically measured enzymatically (Cobas 8000 analyser; Roche Diagnostics). HDL-C was measured using a competitive homogeneous assay (Roche Diagnostics), and LDL-C was calculated using the Friedewald equation [26]. These values were also used to calculate the ratio of LDL-C to HDL-C.

### Diabetes and metabolic syndrome classification

Diabetes was classified according to the ADA guidelines in individuals with fasting glucose levels of >6.94 mmol/l and/or HbA_1c_ levels of ≥6.5% (48 mmol/mol) in the absence of known diabetes. Those with self-reported, doctor-diagnosed diabetes were also classified as diabetic. Prediabetes was classified as a fasting glucose level between 5.55 and 6.94 mmol/l and/or an HbA_1c_ level between 5.7% (39 mmol/mol) and 6.4% (46 mmol/mol) [27]. The remaining normal-glycaemic individuals, therefore, had fasting glucose and HbA_1c_ levels of <5.55 mmol/l and <5.7%, respectively. The metabolic syndrome components were assessed as the following: (1) waist circumference >102 cm (men) or >88 cm (women); (2) plasma triacylglycerol >1.70 mmol/l; (3) plasma HDL-C <1.03 mmol/l (men) or <1.29 mmol/l (women); (4) BP ≥130 mmHg (systolic) and/or ≥85 (diastolic) mmHg; and (5) plasma fasted glucose ≥5.55 mmol/l. Each of these components were dichotomised (yes or no) and added together to create a metabolic syndrome component score (range 0–5). Those with a score of ≥3 were classified as having the metabolic syndrome [28].

### Statistical analysis

To approximate a normal distribution of the variables used in the current analyses, we applied transformations based on information criteria obtained from the Ladder-of-Powers in Stata 12 (StataCorp, College Station, TX, USA). The transformation with the least statistical deviation from a normal distribution, indicated by the smallest *χ*^2^ (or the most non-significant *p* value) was used, as previously recommended [29]. Missing data (<7% for all variables) was handled by multiple imputation in IBM SPSS (version 20, Chicago, IL, USA). Briefly, a fully conditional specification method was automatically chosen to replace missing data. In this method, each variable was fitted in a univariate (single dependent variable) model using all other available variables in the model as predictors, and missing values were imputed for each variable being fitted. Linear and logistic regressions were used for continuous and categorical variables, respectively. Relevant variables with already complete data were entered only as predictors to improve estimates. After ten iterations for each of the five imputation datasets, pooled estimates were used for all subsequent analyses below.

First, participant characteristics (i.e. demographics and lifestyle behaviours) were compared between CMV^+^ and CMV^−^ individuals. Student’s *t* tests and *χ*^2^ analyses were used for continuous and categorical variables, respectively.

Second, differences in CMV status by HbA_1c_ and fasting glucose levels and diabetic status were explored using binary logistic regressions. CMV status was entered as the dependent variable, and each factor was entered, in turn, as an independent variable. Potential confounders known to impact CMV infection and reactivation, including age, sex, marital status, SES (job status, manual occupation), and lifestyle factors (smoking, alcohol intake, BMI, and physical activity) [30–32], were statistically controlled in hierarchical models (Models 1–3): Model 1 was adjusted for age and sex; Model 2 was Model 1 additionally adjusted for marital status and SES (job status and manual occupation); and Model 3 was Model 2 further adjusted for smoking, alcohol, BMI and physical activity. These models were entered stepwise as covariates throughout the remaining analyses.

Third, numbers of CD8^+^ EM and EMRA T cells were compared between levels of glycaemic control (indicated by diabetic classification) using ANOVA and ANCOVA. These analyses were stratified by CMV status and the abovementioned potential confounders were entered as covariates (Models 1–3).

Finally, separate linear regressions were used to explore the individual associations of HbA_1c_ and fasting glucose levels with EM and EMRA T cell subset numbers. Potential confounders were entered as covariates using the same models as above.

The above analyses were repeated with each of the dyslipidaemia and CVD risk factors (i.e. total cholesterol, LDL-C, HDL-C, the LDL-C to HDL-C ratio and triacylglycerol) entered separately as independent predictors of EM and EMRA T cell subset numbers. For significant associations, HbA_1c_ was added as a potential mediator to examine the role of glucose levels on lipid metabolism. All analyses were performed with IBM SPSS version 20.

## Results

### Participant characteristics

As shown in Table 1, 400 of the 1,103 (36.3%) participants were CMV^+^. On average, CMV^+^ participants tended to be older and female. They were also more likely to be current or former smokers, to drink less frequently, and to have a lower SES (low job status, more manual occupations and shift work). There was no difference in the amount of cigarettes smoked (among smokers), BMI, WHR or physical activity (*p* > 0.10 for all; Table 1). The tabulation of metabolic risk factors revealed that 290 (26.3%) individuals met the criteria for metabolic syndrome classification. Regarding diabetes, 663 were classified as normal, 404 as prediabetic and 36 as diabetic. Because of the small number of diabetic individuals, the diabetic group was merged with the prediabetic group and labelled ‘hyperglycaemic’.

### Glycaemic control and dyslipidaemia factors are associated with CMV infection

Unadjusted analyses showed that CMV^+^ individuals were more likely to have higher levels of HbA_1c_ (38.1 vs 37.7 mmol/mol) and to be classified as hyperglycaemic, i.e. prediabetic or diabetic (46.2% vs 36.4%; Table 2). In binary logistic regressions, the associations of HbA_1c_ and hyperglycaemic status with CMV infection status were reduced to non-significance after adjusting for age and sex (Model 1) and sociodemographic factors (Model 2), respectively.

**Table 2:** Participant metabolic characteristics

Characteristic	Total	CMV status		*p* value
		Positive	Negative	
*n* (%)	1,103 (100)	400 (36)	703 (64)	
HbA_1c_ (%)	5.61 ± 0.01	5.64 ± 0.02	5.60 ± 0.01	0.047
HbA_1c_ (mmol/mol)	37.84 ± 0.11	38.13 ± 0.18	37.67 ± 0.13	0.060
Fasting glucose (mmol/l)	4.82 ± 0.02	4.85 ± 0.03	4.80 ± 0.02	0.168
Total cholesterol (mmol/l)	5.25 ± 0.03	5.28 ± 0.05	5.24 ± 0.04	0.591
LDL-C (mmol/l)	3.20 ± 0.03	3.22 ± 0.04	3.19 ± 0.03	0.580
HDL-C (mmol/l)	1.40 ± 0.01	1.38 ± 0.02	1.41 ± 0.02	0.068
LDL-C/HDL-C ratio	2.47 ± 0.03	2.54 ± 0.05	2.42 ± 0.04	0.140
Triacylglycerol (mmol/l)	1.42 ± 0.03	1.47 ± 0.05	1.39 ± 0.03	0.145
Diabetes classification (%)	–	–	–	0.005
Normal	60.1	53.9	63.6	–
Prediabetes	36.6	41.9	33.7	–
Diabetes	3.3	4.3	2.7	–
Metabolic syndrome (% yes)	26.3	28.0	25.3	0.368
Waist circumference (% yes)	52.1	54.0	51.0	0.387
Triacylglycerol (% yes)	31.8	35.0	30.0	0.101
HDL-C (% yes)	17.7	21.0	15.8	0.036
BP (% yes)	52.5	49.5	54.2	0.150
Fasting glucose (% yes)	10.0	11.0	9.4	0.442
BMI, (kg/m^2^)	24.46 ± 4.10	24.46 ± 4.13	24.46 ± 3.98	0.929
WHR	0.90 ± 0.08	0.90 ± 0.08	0.90 ± 0.07	0.190

All are unadjusted comparisons of participant metabolic characteristics: a Student’s *t* test was performed for continuous variables and a *χ* ^2^ test for categorical variables. HDL-C is natural log-transformed, LDL-C is square root-transformed and total cholesterol is square root-transformed

Values are means ± SD unless otherwise stated

Unadjusted comparisons of dyslipidaemia factors between CMV^+^ and CMV^−^ individuals revealed that CMV^+^ individuals were also more likely to have HDL-C levels that fell within the metabolic syndrome classification range (men <1.03 mmol/l, women <1.29 mmol/l). However, none of the other metabolic characteristics, including the metabolic syndrome classification, differed by CMV status in unadjusted analyses (Table 2). After progressive adjustment for possible confounders (Models 1–3), the association with the low HDL-C category was no longer significant, but increased levels of continuous HDL-C became significantly associated with CMV infection ((OR 0.55 [95% CI] 0.319–0.960), *p* = 0.035). That is, individuals with higher HDL-C levels (natural log-transformed) had a lower risk of CMV infection.

### Glycaemic control and its interaction with CMV infection are associated with dCTL numbers

**Fig. 1:**
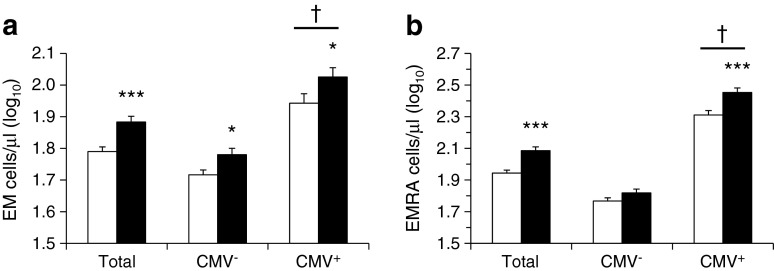
Unadjusted comparison of (**a**) EM (CD27^−^CD45RA^−^) and (**b**) EMRA (CD27^−^CD45RA^+^) CD8^+^ T cell subset numbers by glycaemic status and CMV infection. White bars, normoglycaemic; black bars, hyperglycaemic. *p < 0.05 and ***p < 0.001 represent levels of significant difference from normoglycaemic. ^†^ *p* < 0.001 represents significant difference from CMV^−^

Fig. 1 shows the unadjusted comparisons of EM and EMRA T cell numbers stratified by glycaemic status and CMV infection. For all participants, individuals classified as hyperglycaemic had 26.6% higher numbers of EM (110.2 vs 87.0 cells/μl) and 41.2% higher EMRA T cells (218.1 vs 154.5 cells/μl; both *p* < 0.001) than normoglycaemic participants.

When further stratified by CMV status, the results showed significantly more EMRA T cells in hyperglycaemic vs normoglycaemic CMV^+^ individuals (*p* < 0.001), while no such difference was observed in the CMV^−^ group (Fig. 1). This effect remained significant after full adjustment, and was accompanied by a significant interaction of CMV status by glycaemic status with EMRA levels (*p* = 0.031). In addition, these effects did not appear to be caused by the small group of diabetic individuals because identical results were found when diabetic participants were excluded from the analyses (data not shown). Analyses adjusted for age and sex showed that there were more EM T cells in hyperglycaemic individuals than in the normoglycaemic group, but this did not reach significance.

### Increased HbA_1c_ is associated with increased dCTL numbers

Table 3 shows the linear associations of metabolic factors with dCTL numbers. Overall, higher levels of continuous HbA_1c_ were associated with increased numbers of dCTLs (EM: *B* = 1.87, *p* < 0.001; EMRA: *B* = 2.05, *p* < 0.01) after adjustment for age, sex, marital status and SES (i.e. job status and manual occupation; Model 2). These associations remained significant after additional adjustment for lifestyle factors (Model 3; both *p* < 0.05). The fasting glucose level was nonsignificantly associated with dCTL numbers in the overall sample (Table 3). When analyses were stratified by CMV status, the relationship between HbA_1c_ and increased dCTL numbers remained in CMV^+^, but not in CMV^−^ individuals (both *p* < 0.01).

**Table 3:** Unstandardised coefficients from linear regressions of metabolic factors and EM and EMRA T cell numbers

Variables	EM (CD27^−^CD45RA^−^)			EMRA (CD27^−^CD45RA^+^)		
	Model 1	Model 2	Model 3	Model 1	Model 2	Model 3
All participants (*n* = 1,103)						
HbA_1c_	2.03 (0.58)***	1.87 (0.59)***	1.54 (0.59)*	2.20 (0.77)**	2.05 (0.77)**	1.68 (0.78)*
Fasting glucose	2.15 (2.06)	2.31 (2.06)	3.23 (2.07)	−2.92 (2.69)	−2.74 (2.70)	−1.72 (2.73)
Cholesterol	0.01 (0.01)	0.01 (0.01)	0.01 (0.01)	0.01 (0.01)	0.01 (0.01)	0.02 (0.01)
LDL-C	0.01 (0.01)	0.01 (0.01)	0.01 (0.01)	0.01 (0.01)	0.01 (0.01)	0.01 (0.01)
HDL-C	−0.13 (0.05)**	−0.13 (0.05)**	−0.06 (0.05)	−0.16 (0.06)**	−0.15 (0.06)*	−0.08 (0.06)
LDL-C/HDL-C	0.20 (0.07)**	0.20 (0.07)**	0.14 (0.07)*	0.21 (0.09)*	0.21 (0.09)*	0.13 (0.10)
Triacylglycerol	0.14 (0.05)**	0.13 (0.05)**	0.11 (0.05)*	0.21 (0.07)**	0.20 (0.07)**	0.18 (0.07)*
CMV negative (*n* = 703)						
HbA_1c_	0.97 (0.65)	0.86 (0.65)	0.46 (0.66)	0.87 (0.23)	0.94 (0.82)	0.48 (0.84)
Fasting glucose	2.34 (2.18)	2.40 (2.18)	3.75 (2.23)	−1.3 (2.77)	−1.29 (2.77)	0.36 (2.84)
Cholesterol	0.00 (0.01)	0.00 (0.01)	0.00 (0.01)	0.01 (0.01)	0.01 (0.01)	0.01 (0.01)
LDL-C	0.00 (0.01)	0.01 (0.01)	0.00 (0.01)	0.01 (0.01)	0.01 (0.01)	0.01 (0.01)
HDL-C	−0.09 (0.05)	−0.08 (0.05)	−0.03 (0.05)	−0.02 (0.06)	−0.03 (0.06)	0.02 (0.07)
LDL-C/HDL-C	0.11 (0.07)	0.12 (0.07)	0.06 (0.08)	0.08 (0.09)	0.09 (0.09)	0.01 (0.10)
Triacylglycerol	0.07 (0.06)	0.06 (0.06)	0.03 (0.06)	0.12 (0.07)	0.13 (0.07)	0.09 (0.07)
CMV positive (*n* = 400)						
HbA_1c_	3.11 (1.03)**	3.02 (1.03)**	2.75 (1.03)**	3.02 (1.00)**	2.92 (1.01)**	2.90 (1.02)**
Fasting glucose	2.41 (3.82)	2.19 (3.83)	2.36 (3.83)	−3.86 (3.72)	−3.78 (3.74)	−3.40 (3.77)
Cholesterol	0.04 (0.02)*	0.04 (0.02)*	0.04 (0.02)*	0.03 (0.02)	0.03 (0.02)	0.03 (0.02)
LDL-C	0.03 (0.01)	0.03 (0.01)	0.03 (0.01)*	0.02 (0.01)	0.02 (0.01)	0.02 (0.01)
HDL-C	−0.11 (0.08)	−0.10 (0.08)	−0.04 (0.09)	−0.11 (0.08)	−0.10 (0.08)	−0.06 (0.09)
LDL-C/HDL-C	0.27 (0.12)*	0.26 (0.12)*	0.22 (0.13)	0.21 (0.12)	0.22 (0.12)	0.18 (0.13)
Triacylglycerol	0.22 (0.09)*	0.22 (0.09)*	0.20 (0.10)*	0.23 (0.09)*	0.22 (0.09)*	0.21 (0.10)*

Data are unstandardised coefficients (B) (SEM)

Model 1: adjusted for age and sex

Model 2: model 1 with additional adjustment for marital status and SES (job status and manual occupation)

Model 3: model 2 further adjusted for smoking, alcohol, BMI and physical activity

*p < 0.05, **p < 0.01 and ***p = 0.001

### CMV^−^ and HbA_1c_-related associations between dyslipidaemia factors and dCTL numbers

For all participants, linear regression showed that higher continuous levels of triacylglycerol were associated with increased numbers of dCTLs after adjustment for confounders (Models 1–3; Table 3). Lower HDL-C levels and a higher LDL-C/HDL-C ratio were also significantly associated with increased EM and EMRA numbers after adjustment for sociodemographic factors (Model 2). Except for the LDL-C/HDL-C association with EM T cells, these relationships were attenuated by additional adjustment for lifestyle factors (Model 3). Total cholesterol and LDL-C levels were nonsignificantly associated with dCTL numbers in the full sample (Table 3).

When analyses were stratified by CMV status, the relationship between triacylglycerol and increased dCTL numbers was found only in CMV^+^ individuals. Additionally, in the CMV^+^ group, total cholesterol and the LDL-C/HDL-C ratio were positively associated with EM T cell numbers after adjustment for sociodemographic factors (Model 2; both *p* < 0.05), although only total cholesterol remained significant after full adjustment (Model 3; Table 3).

To test the influence of glucose metabolism on the significant lipid profile associations in CMV^+^ individuals (i.e. triacylglycerol and cholesterol), HbA_1c_ was additionally entered into each of these fully adjusted models. After the addition of HbA_1c_ to the model, the associations between triacylglycerol and dCTL were attenuated, while the relationship between cholesterol and EM T cells remained significant (data not shown). Among CMV^−^ individuals, no significant associations were observed between any metabolic factors and dCTL numbers (Table 3).

## Discussion

A hallmark of an ageing immune system is the accumulation of differentiated CD8^+^ T cells, which is strongly enhanced in CMV-infected individuals. The present study demonstrated that impaired glycaemic control, as measured by HbA_1c_ levels, is associated with elevated numbers of dCTL in CMV^+^ individuals, but not in CMV^−^ individuals. This association was robust to adjustment for demographic, SES and lifestyle factors. Thus, this study is the first to provide evidence that glycaemic control may contribute to immunosenescence by amplifying the effects of CMV on T cell differentiation. This mechanism may contribute to the impaired immunity seen in hyperglycaemia and diabetic patients, and may possibly be a pathway linking CMV to increased CVD risk.

HbA_1c_ may be positively associated with dCTL in CMV^+^ individuals due to a synergistic impact on both the extent of CMV replication and the T cell response to CMV activity. Elevated glucose levels may: (1) directly and indirectly increase the efficiency and frequency of CMV replication; and (2) enhance the T cell responses to CMV. First, CMV-induced upregulation of GLUT4 enhances glucose uptake [33]. This excess glucose influx is diverted towards the biosynthesis of fatty acids, which are used to directly increase viral production and enhance infectivity [34]. Indirectly, elevated glucose promotes the production of reactive oxygen species, which are known to stimulate the CMV promoter region and provide the first step that is necessary, but not sufficient, for the reactivation of CMV [35, 36]. Second, evidence from in vitro studies demonstrates that strong, repeated antigen receptor stimulation (e.g. by CMV) can lead to the upregulation of GLUT1 and to enhanced glucose uptake by T cells. Excess glucose uptake was shown to parallel increased T cell activation, proinflammatory cytokine production and an elevated threshold for cell death [21, 22]. Although not directly investigated in the present study, these represent biologically plausible mechanisms through which elevated glucose could augment dCTL numbers via CMV activity.

Attenuation of the relationship between hyperglycaemia and CMV infection by sociodemographic factors is in line with the finding of null associations after similar adjustments in other studies [37, 38], but is inconsistent with the somewhat divergent epidemiological data from Chen and colleagues in very old (85 years) adults [39]. The former finding suggests that unadjusted differences in glucose by CMV status are likely to be a consequence of common predisposing factors, rather than being causally linked. Indeed, both CMV infection and dysregulated glucose metabolism are more common in individuals with a variety of pre-existing health risk factors. For example, CMV has a non-random distribution in the population: infection is particularly prevalent among those who are typically older, current smokers or have low SES (job status, education) [31, 40]. On the other hand, the robust linear relationships between HbA_1c_ and both EM and EMRA T cells found only in CMV^+^ individuals suggest that glycaemic status may contribute to immune responses to CMV reactivation rather than to initial infection.

The current positive relationship between markers of dyslipidaemia and numbers of dCTLs in CMV^+^ individuals appears to be partly a by-product of the intrinsic link between HbA_1c_ level and lipid metabolism found in both diabetic [23] and non-diabetic adults [41]. Evidence for this relationship is provided by the null associations between triacylglycerol and dCTL numbers in CMV^+^ individuals after additional adjustment for HbA_1c_ level in the current study. In contrast, the cholesterol–EM association remained after additional adjustment for HbA_1c_ level, making a more direct effect of CMV more plausible. The factors underlying this association are not clear, but could reflect the manifestation of a host defence mechanism to limit CMV infectivity. At an early stage of CMV infection, cells increase the expression of CD91, which regulates lipid metabolism and decreases intracellular cholesterol [42]. As intracellular cholesterol is necessary for enhanced CMV virus production and effective entry into other cells [43, 44], a reduction in cellular cholesterol uptake by the host cell could explain the association between elevated cholesterol and EM accumulation seen here [42]. This does not, however, rule out the possibility of CMV-induced alterations in systemic lipid and glucose levels.

The current finding that CMV^+^ individuals with higher levels of HbA_1c_ and CVD risk factors (cholesterol and triacylglycerol) had elevated dCTLs provides further evidence for a common impact of both CMV [30, 45, 46] and dysregulated glucose metabolism [47] on cardiac health. Consistent with this notion, studies have reported associations among circulating dCTL, CMV-specific T cell responses and increased CVD risk factors, such as heart valve calcification, carotid artery thickness and increased BP [48–50]. Taken together, these findings support the hypothesis proposed by Simanek and colleagues [32] that CMV infection and inflammation partially impact mortality risk via their combined contribution to other CVD risk factors, and further suggest dysregulated glucose metabolism and increased dCTLs as additional mechanisms.

There are a number of limitations with the current study that should be acknowledged. First, the low number of diabetic participants precluded their inclusion as a separate group; comparisons were instead performed between normoglycaemic and a merged hyperglycaemic group. Information about whether these participants had type 1 or type 2 diabetes was also unavailable; however, in the general population, type 1 diabetes represents only 5–10% of the diabetes cases [27]. Likewise, clinically relevant markers of immunosenescence (e.g. short telomere length) are observed even at the early stages of glucose dysregulation [4, 20], and the associations found in hyperglycaemic individuals were not altered by removal of the diabetic group from the analyses. Second, there was no direct measure of subsequent CMV reactivation. Nevertheless, the selective EMRA T cell accumulation is almost exclusively associated with CMV infection, and there is a strong empirical basis to suggest that these associations reflect CMV activity [2, 6, 9]. However, future studies should include more direct measures of CMV reactivation (e.g. quantitative CMV-specific antibody levels) for comparison. Finally, because of the cross-sectional nature of the study, we are unable to discern cause–effect relationships. Given the complex interplay between components of the immune system, metabolic factors, CMV and ageing, as well as the contribution of potential intermediaries such as oxidative stress and inflammation to these processes, bidirectional or cyclical relationships between these factors cannot be ruled out.

In conclusion, we observed associations between measures of glucose metabolism (i.e. HbA_1c_ level and diabetic status), dyslipidaemia (total cholesterol and triacylglycerol levels) and dCTL subsets in CMV^+^ individuals. These associations with HbA_1c_ level withstood adjustment for demographic, SES and lifestyle factors known to impact both CMV infection and glucose metabolism, thus demonstrating a robust association. Overall, it appears that these metabolic factors act reciprocally with CMV to amplify the accumulation of EM and EMRA CD8^+^ T cells, and represent potentially biologically relevant pathways underlying the CMV-induced acceleration of immunosenescence. These data also highlight CMV and dCTL accumulation as a potentially overlooked mechanism underlying the associations of hyperglycaemia and diabetes with impaired immunity. These links to immunosenescence are particularly relevant in the context of increased incidence of type 2 diabetes and an associated defective viral response in an ageing population.

## Electronic supplementary material

ESM Fig. 1 (PDF 4,785 kb)

## Abbreviations

APC: Allophycocyanin
CMV: Cytomegalovirus
CVD: Cardiovascular disease
Cy7: Cyanine dye 7
dCTL: Differentiated cytotoxic T cell
EM: Effector memory
EMRA: CD45RA re-expressing effector memory
HDL-C: HDL-cholesterol
LDL-C: LDL-cholesterol
PE: Phycoerythrin
SES: Socioeconomic status

## References

[1] Larbi A, Franceschi C, Mazzatti D, Solana R, Wikby A, Pawelec G (2008). Aging of the immune system as a prognostic factor for human longevity. Physiology.

[2] Turner JE, Campbell JP, Edwards KM (2014). Rudimentary signs of immunosenescence in Cytomegalovirus-seropositive healthy young adults. Age.

[3] Franceschi C, Bonafè M, Valensin S (2000). Inflamm-aging. An evolutionary perspective on immunosenescence. Ann N Y Acad Sci.

[4] Salpea KD, Humphries SE (2010). Telomere length in atherosclerosis and diabetes. Atherosclerosis.

[5] Sansoni P, Vescovini R, Fagnoni F (2008). The immune system in extreme longevity. Exp Gerontol.

[6] Chidrawar S, Khan N, Wei W (2009). Cytomegalovirus-seropositivity has a profound influence on the magnitude of major lymphoid subsets within healthy individuals. Clin Exp Immunol.

[7] Dowd JB, Bosch JA, Steptoe A (2013). Cytomegalovirus is associated with reduced telomerase activity in the Whitehall II cohort. Exp Gerontol.

[8] van de Berg PJ, Griffiths SJ, Yong SL (2010). Cytomegalovirus infection reduces telomere length of the circulating T cell pool. J Immunol.

[9] van de Berg PJ, van Stijn A, Ten Berge IJ, van Lier RA (2008). A fingerprint left by cytomegalovirus infection in the human T cell compartment. J Clin Virol.

[10] Kuijpers TW, Vossen MT, Gent MR (2003). Frequencies of circulating cytolytic, CD45RA+CD27-, CD8+ T lymphocytes depend on infection with CMV. J Immunol.

[11] Messaoudi I, Lemaoult J, Guevara-Patino JA, Metzner BM, Nikolich-Zugich J (2004). Age-related CD8 T cell clonal expansions constrict CD8 T cell repertoire and have the potential to impair immune defense. J Exp Med.

[12] Macaulay R, Akbar AN, Henson SM (2013). The role of the T cell in age-related inflammation. Age.

[13] Akbar AN, Henson SM (2011). Are senescence and exhaustion intertwined or unrelated processes that compromise immunity?. Nat Rev Immunol.

[14] Almanzar G, Schwaiger S, Jenewein B (2005). Long-term cytomegalovirus infection leads to significant changes in the composition of the CD8+ T-cell repertoire, which may be the basis for an imbalance in the cytokine production profile in elderly persons. J Virol.

[15] Allard R, Leclerc P, Tremblay C, Tannenbaum TN (2010). Diabetes and the severity of pandemic influenza A (H1N1) infection. Diabetes Care.

[16] Joshi N, Caputo GM, Weitekamp MR, Karchmer AW (1999). Infections in patients with diabetes mellitus. N Engl J Med.

[17] Shah BR, Hux JE (2003). Quantifying the risk of infectious diseases for people with diabetes. Diabetes Care.

[18] Egawa Y, Ohfuji S, Fukushima W (2014). Immunogenicity of influenza A(H1N1)pdm09 vaccine in patients with diabetes mellitus: with special reference to age, body mass index, and HbA1c. Hum Vaccin Immunother.

[19] Esposito K, Nappo F, Marfella R (2002). Inflammatory cytokine concentrations are acutely increased by hyperglycemia in humans: role of oxidative stress. Circulation.

[20] Adaikalakoteswari A, Balasubramanyam M, Ravikumar R, Deepa R, Mohan V (2007). Association of telomere shortening with impaired glucose tolerance and diabetic macroangiopathy. Atherosclerosis.

[21] Maciver NJ, Jacobs SR, Wieman HL, Wofford JA, Coloff JL, Rathmell JC (2008). Glucose metabolism in lymphocytes is a regulated process with significant effects on immune cell function and survival. J Leukoc Biol.

[22] Zhao Y, Altman BJ, Coloff JL (2007). Glycogen synthase kinase 3alpha and 3beta mediate a glucose-sensitive antiapoptotic signaling pathway to stabilize Mcl-1. Mol Cell Biol.

[23] Khan HA, Sobki SH, Khan SA (2007). Association between glycaemic control and serum lipids profile in type 2 diabetic patients: HbA1c predicts dyslipidaemia. Clin Exp Med.

[24] Jönsson D, Rosengren A, Dotevall A, Lappas G, Wilhelmsen L (1999). Job control, job demands and social support at work in relation to cardiovascular risk factors in MONICA 1995, Göteborg. J Cardiovasc Risk.

[25] Sacks DB, Bruns DE, Goldstein DE, Maclaren NK, McDonald JM, Parrott M (2002). Guidelines and recommendations for laboratory analysis in the diagnosis and management of diabetes mellitus. Clin Chem.

[26] Friedewald WT, Levy RI, Fredrickson DS (1972). Estimation of the concentration of low-density lipoprotein cholesterol in plasma, without use of the preparative ultracentrifuge. Clin Chem.

[27] American Diabetes A (2013). Diagnosis and classification of diabetes mellitus. Diabetes Care.

[28] Grundy SM, Cleeman JI, Daniels SR (2005). Diagnosis and management of the metabolic syndrome: an American Heart Association/National Heart, Lung, and Blood Institute Scientific Statement. Circulation.

[29] Gould W, Hilbe J (1991) sed2. Ladder-of-Powers variable transformation. Stata Technical Bulletin July:14-15

[30] Savva GM, Pachnio A, Kaul B (2013). Cytomegalovirus infection is associated with increased mortality in the older population. Aging Cell.

[31] Rector JL, Dowd JB, Loerbroks A (2014). Consistent associations between measures of psychological stress and CMV antibody levels in a large occupational sample. Brain Behav Immun.

[32] Simanek AM, Dowd JB, Pawelec G, Melzer D, Dutta A, Aiello AE (2011). Seropositivity to cytomegalovirus, inflammation, all-cause and cardiovascular disease-related mortality in the United States. PLoS One.

[33] Yu Y, Maguire TG, Alwine JC (2011). Human cytomegalovirus activates glucose transporter 4 expression to increase glucose uptake during infection. J Virol.

[34] Spencer CM, Schafer XL, Moorman NJ, Munger J (2011). Human cytomegalovirus induces the activity and expression of acetyl-coenzyme a carboxylase, a fatty acid biosynthetic enzyme whose inhibition attenuates viral replication. J Virol.

[35] Cohen G, Riahi Y, Shamni O (2011). Role of lipid peroxidation and PPAR- in amplifying glucose-stimulated insulin secretion. Diabetes.

[36] Jaganjac M, Matijevic T, Cindric M (2010). Induction of CMV-1 promoter by 4-hydroxy-2-nonenal in human embryonic kidney cells. Acta Biochim Pol.

[37] Haeseker MB, Pijpers E, Dukers-Muijrers NH (2013). Association of cytomegalovirus and other pathogens with frailty and diabetes mellitus, but not with cardiovascular disease and mortality in psycho-geriatric patients; a prospective cohort study. Immun Ageing.

[38] Lutsey PL, Pankow JS, Bertoni AG, Szklo M, Folsom AR (2009). Serological evidence of infections and type 2 diabetes: the MultiEthnic Study of Atherosclerosis. Diabet Med.

[39] Chen S, de Craen AJ, Raz Y (2012). Cytomegalovirus seropositivity is associated with glucose regulation in the oldest old. Results from the Leiden 85-plus Study. Immun Ageing.

[40] Dowd JB, Aiello AE, Alley DE (2009). Socioeconomic disparities in the seroprevalence of cytomegalovirus infection in the US population: NHANES III. Epidemiol Infect.

[41] Selvin E, Steffes MW, Zhu H (2010). Glycated hemoglobin, diabetes, and cardiovascular risk in nondiabetic adults. N Engl J Med.

[42] Gudleski-O'Regan N, Greco TM, Cristea IM, Shenk T (2012). Increased expression of LDL receptor-related protein 1 during human cytomegalovirus infection reduces virion cholesterol and infectivity. Cell Host Microbe.

[43] Chukkapalli V, Heaton NS, Randall G (2012). Lipids at the interface of virus-host interactions. Curr Opin Microbiol.

[44] Williamson CD, Zhang A, Colberg-Poley AM (2011). The human cytomegalovirus protein UL37 exon 1 associates with internal lipid rafts. J Virol.

[45] Gkrania-Klotsas E, Langenberg C, Sharp S, Luben R, Khaw K-T, Wareham N (2012). Higher immunoglobulin G antibody levels against cytomegalovirus are associated with incident ischemic heart disease in the population-based EPIC-Norfolk cohort. J Infect Dis.

[46] van de Berg PJ, Yong S-L, Remmerswaal EB, van Lier RA, ten Berge IJ (2012). Cytomegalovirus-induced effector T cells cause endothelial cell damage. Clin Vaccine Immunol.

[47] Barr EL, Boyko EJ, Zimmet PZ, Wolfe R, Tonkin AM, Shaw JE (2009). Continuous relationships between non-diabetic hyperglycaemia and both cardiovascular disease and all-cause mortality: the Australian Diabetes, Obesity, and Lifestyle (AusDiab) study. Diabetologia.

[48] Hsue PY, Hunt PW, Sinclair E (2006). Increased carotid intima-media thickness in HIV patients is associated with increased cytomegalovirus-specific T-cell responses. Aids.

[49] Terrazzini N, Bajwa M, Vita S (2014). A novel cytomegalovirus-induced regulatory-type T-cell subset increases in size during older life and links virus-specific immunity to vascular pathology. J Infect Dis.

[50] Winchester R, Wiesendanger M, O'Brien W (2011). Circulating activated and effector memory T cells are associated with calcification and clonal expansions in bicuspid and tricuspid valves of calcific aortic stenosis. J Immunol.

