# Supplementary material for: Elevated HbA1c levels and the accumulation of differentiated T cells in CMV+ individuals
Source: Diabetologia. 2015 Aug 20;58(11):2596–605. doi: 10.1007/s00125-015-3731-4 (PMC4589544; doi:10.1007/s00125-015-3731-4)
Supplement: Supplementary file 1 — (PDF 4,785 kb) [file 125_2015_3731_MOESM1_ESM.pdf]

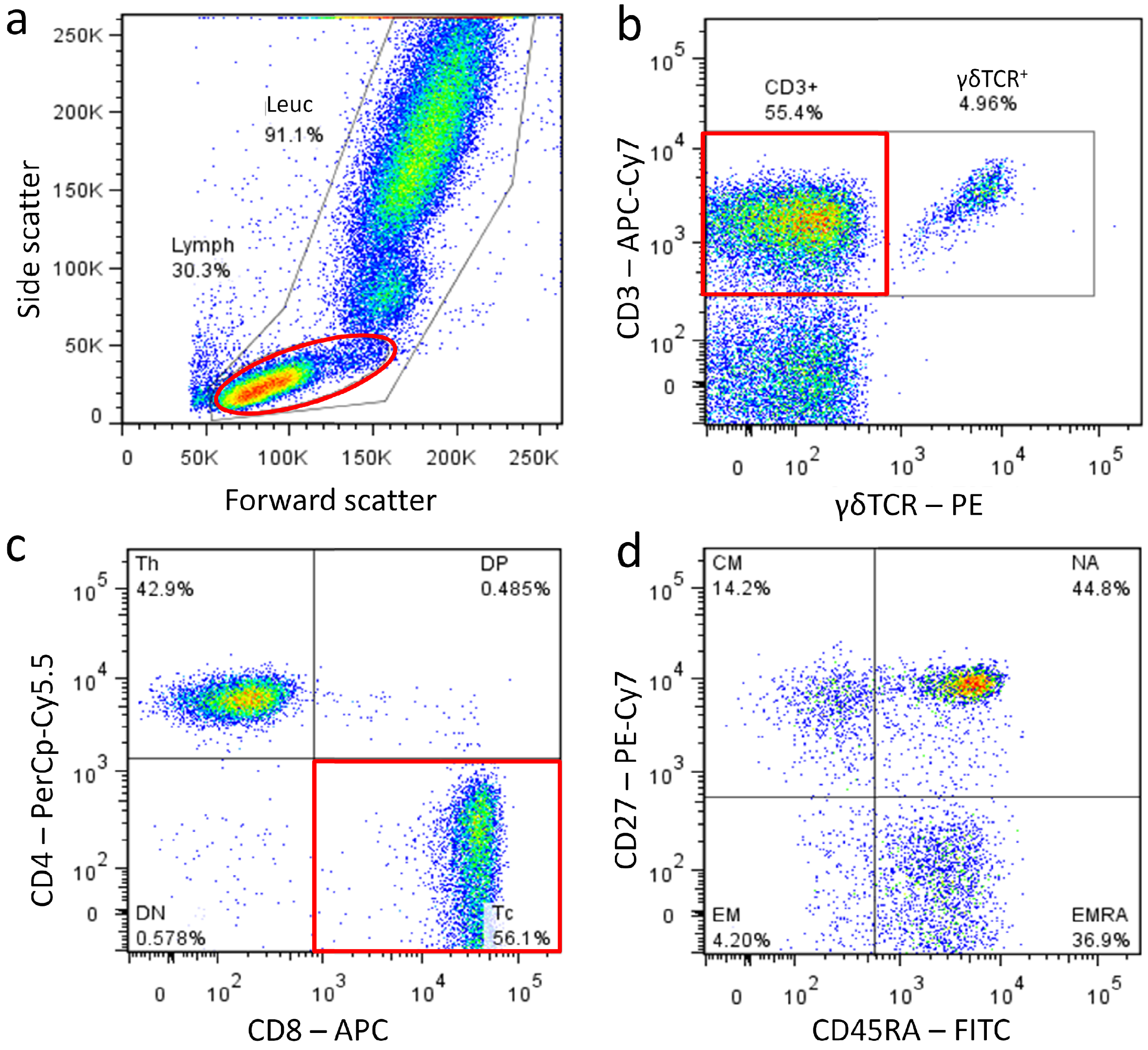

ESM Figure 1: Representative plots of the gating strategy utilized in the analysis of flow cytometry data from whole blood. (a) The forward scatter versus side scatter plot, with at least 50,000 recorded events in the lymphocyte (lymph) gate, was used to gate all leucocytes (leuc), excluding the majority of debris. The lymphocyte percentage was calculated relative to the leucocyte gate. (b) Next, $\gamma\delta TCR^-CD3^+$ T-cells were identified. (c) Among $\gamma\delta TCR^-CD3^+$ T-cells, the single positive $CD8^+$ T-cells were selected for further analysis. (d) $CD8^+$ T-cells were further sub-divided into naïve (NA; $CD45RA^+CD27^+$), central memory (CM; $CD45RA^-CD27^+$), effector memory (EM; $CD45RA^-CD27^-$) and CD45RA re-expressing effector memory (EMRA; $CD45RA^+CD27^-$) subsets. DP, double positive; DN, double negative; Th, CD4 single positive; Tc, CD8 single positive.
